# Chromosomes of Asian cyprinid fishes: cytogenetic analysis of two representatives of small paleotetraploid tribe Probarbini

**DOI:** 10.1186/s13039-018-0399-8

**Published:** 2018-09-04

**Authors:** Pasakorn Saenjundaeng, Marcelo de Bello Cioffi, Ezequiel Aguiar de Oliveira, Alongklod Tanomtong, Weerayuth Supiwong, Sumalee Phimphan, Maria João Collares-Pereira, Alexandr Sember, Luiz Antonio Carlos Bertollo, Thomas Liehr, Cassia Fernanda Yano, Terumi Hatanaka, Petr Ráb

**Affiliations:** 1 Toxic Substances in Livestock and Aquatic Animals Research Group, Department of Biology, Faculty of Science, Khon Kaen University, Muang District, Khon Kaen, Thailand; 2 Departamento de Genética e Evolução, Universidade Federal de São Carlos, São Carlos, SP Brazil; 3 Secretaria de Estado de Educação de Mato Grosso – SEDUC-MT, Cuiabá, MT Brazil; 4 Faculty of Applied Science and Engineering, Khon Kaen University, Nong Kai Campus, Muang, Nong Kai Thailand; 5 Faculdade de Ciencias, Centre for Ecology, Evolution and Environmental Changes, Universidade de Lisboa, Campo Grande, PT–1749-016 Lisbon, Portugal; 6 Laboratory of Fish Genetics, Institute of Animal Physiology and Genetics, Czech Academy of Sciences, Rumburská 89, 277 21 Liběchov, Czech Republic; 7 Institute of Human Genetics, Jena University Hospital, Am Klinikum 1, D-07747 Jena, Germany

**Keywords:** Fish cytogenetics, Cyprinidae, Chromosomal markers, rDNAs, Microsatellites

## Abstract

**Background:**

Polyploidy, although still poorly explored, represents an important evolutionary event in several cyprinid clades. Herein, *Catlocarpio siamensis* and *Probarbus jullieni* - representatives of the paleotetraploid tribe Probarbini, were characterized both by conventional and molecular cytogenetic methods.

**Results:**

Alike most other paleotetraploid cyprinids (with 2n = 100), both species studied here shared 2n = 98 but differed in karyotypes: *C. siamensis* displayed 18m + 34sm + 46st/a; NF = 150, while *P. jullieni* exhibited 26m + 14sm + 58st/a; NF = 138. Fluorescence in situ hybridization (FISH) with rDNA probes revealed two (5S) and eight (18S) signals in *C. siamensis*, respectively, and six signals for both probes in *P. jullieni*. FISH with microsatellite motifs evidenced substantial genomic divergence between both species. The almost doubled size of the chromosome pairs #1 in *C. siamensis* and #14 in *P. jullieni* compared to the rest of corresponding karyotypes indicated chromosomal fusions.

**Conclusion:**

Based on our findings, together with likely the same reduced 2n = 98 karyotypes in the remainder Probarbini species, we hypothesize that the karyotype 2n = 98 might represent a derived character, shared by all members of the Probarbini clade. Besides, we also witnessed considerable changes in the amount and distribution of certain repetitive DNA classes, suggesting complex post-polyploidization processes in this small paleotetraploid tribe.

## Background

One of the most important evolutionary characteristics of Teleostei is a lineage-specific polyploidization (i.e. teleost-specific whole-genome duplication = TS-WGD) that occurred approximately 226–316 Mya [1], subsequently to its divergence from the remaining actinopterygians (i.e. bichirs, sturgeons, paddlefishes, gars and bowfin) [2, 3]. Furthermore, additional whole-genome duplications (WGDs) took place independently in several other teleostean lineages, such as, e.g., Catostomidae [4], Cobitidae [5], Callichthyidae [6, 7], Salmoniformes [8] and especially Cyprinidae [9–11].

Independent evolutionary tetraploidy and even recurrent hexaploid events of allopolyploid origin have already been evidenced for several cyprinid clades taxonomically recognized as tribes [9]; these are Probarbini, Torini, Smiliogastrini, Cyprinini, Spinibarbini, Schizothoracini, Schizopygopsini and Barbini and overall account for more than 400 polyploid species. Probarbini represents the most early-diverging group [9], with only two genera: (i) the monotypic *Catlocarpio* (*C. siamensis* Boulenger, 1898) being the largest known cyprinid species attaining a length of up to three meters [12, 13], and (ii) the genus *Probarbus*, with three valid species [14] (*P. jullieni* Sauvage, 1880, *P. labeamajor* Roberts, 1992 and *P. labeaminor* Roberts, 1992). These four potamodromous species from large river systems in Southeast Asia have been and are heavily declining in the sizes of populations due to fishery pressures, but also due to habitat loss and degradation. Thus, since extirpated from most of their native range, these species were considered as “threatened” following IUCN criteria (IUCN Red List of Threatened Species 2012) [15–18].

Irrespective of the evolutionary importance of paleopolyploidy present in various clades of cyprinid fishes [9], its cytogenetic investigation remains practically restricted to reports about diploid chromosome numbers (2n) and karyotype descriptions, rarely complemented with particular chromosome banding protocols [19]. Even less studies applied molecular cytogenetic approaches, such as e.g. the physical mapping of ribosomal genes in Indian species of the genus *Tor*, tribe Torini [20, 21]. The 2n and the karyotypes of the species studied herein were previously reported based exclusively on conventionally Giemsa-stained chromosomes [22, 23]. Based on those reports, two individuals of *P. jullieni* and *C. siamensis*, obtained from aquarium fish dealer, possessed 2n = 98 chromosomes, differing in the proportion of chromosome categories: (18m + 54sm/st + 26a) in *P. jullieni* and (18m + 28sm/st + 52a) in *C. siamensis*. The present study includes in depth cytogenetic analyses of these two species, comprising conventional Giemsa-staining, C-banding and fluorescence in situ hybridization (FISH) approaches with chromosomal mapping of several repetitive DNA classes.

## Results

### Karyotypes

**Fig. 1:**
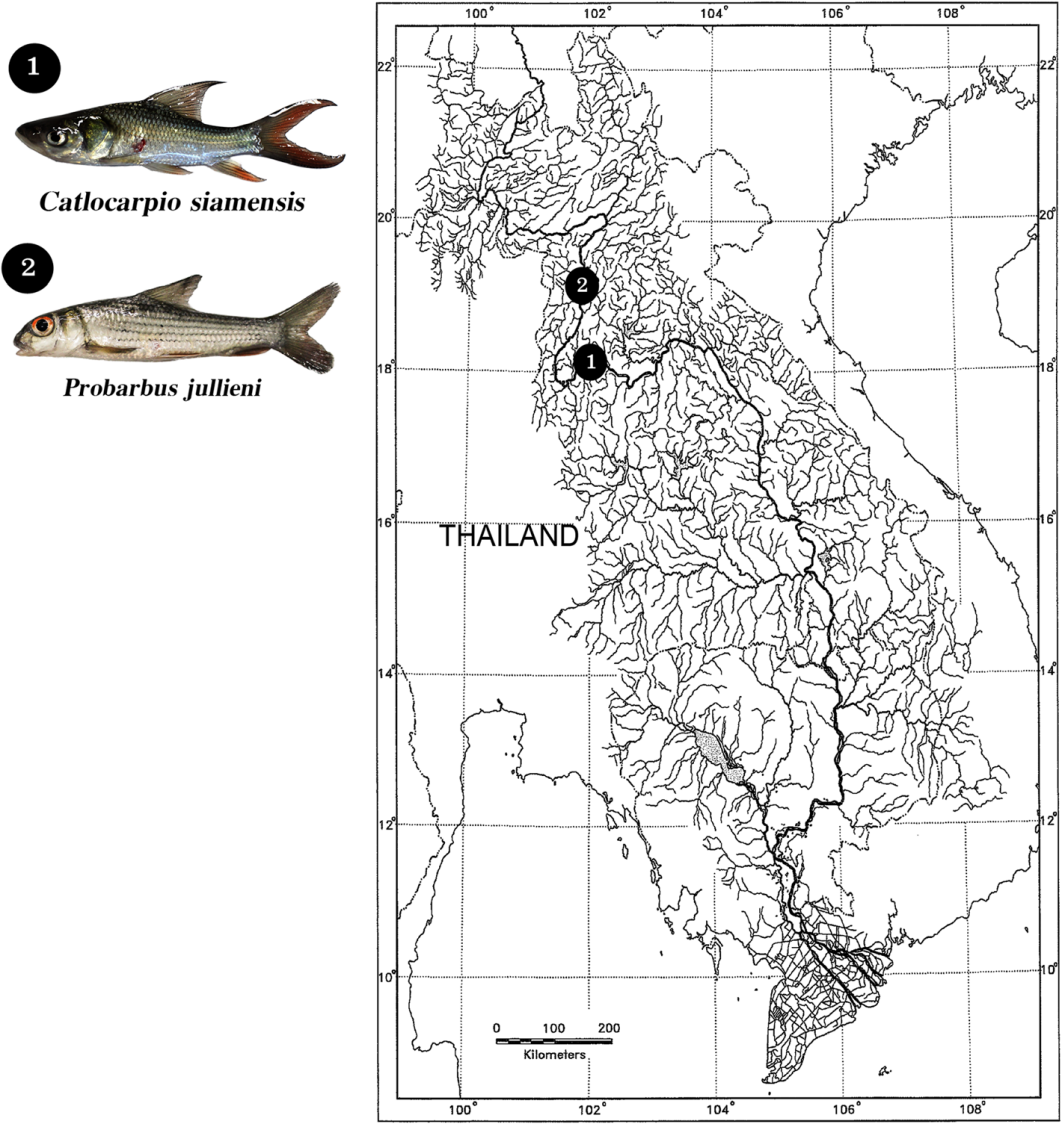
Collection sites of *Catlocarpio siamensis* and *Probarbus jullieni* in Thailand examined in the present study (Map of the Mekong River basin modified from Rainboth [13])

*C. siamensis* possesses 2n = 98 with the karyotype composed of 18m + 34sm + 46st/a elements, with number of chromosomal arms (NF, Nombre Fondamental) being equal to 150. *P. jullieni* shares with the former species 2n = 98, but differs in karyotype composition having 26m + 14sm + 58st/a and NF = 138. The chromosome pairs #1 in *C. siamensis* and #14 in *P. jullieni* were almost doubled in size when compared to the rest of corresponding karyotypes. No differences between male and female karyotypes occur in both species (Fig. 1).

### Chromosome mapping of 5S and 18S ribosomal DNA (rDNA) sequences

In the genome of *C. siamensis*, the minor 5S rDNA class is located on a single chromosomal pair (#27), while the major 18S rDNA occurs in four chromosome pairs (#22, #23, #40, #41). By contrast, in *P. jullieni* the 5S rDNA genes are located on three chromosomal pairs (#23, #24, #25), while the 18S rDNA are in another three ones (#19, #35, #36). Consequently, no co-localization of rDNA classes in the genome of either species was detected (Fig. 1).

### Chromosomal mapping of microsatellite sequences

**Fig. 2:**
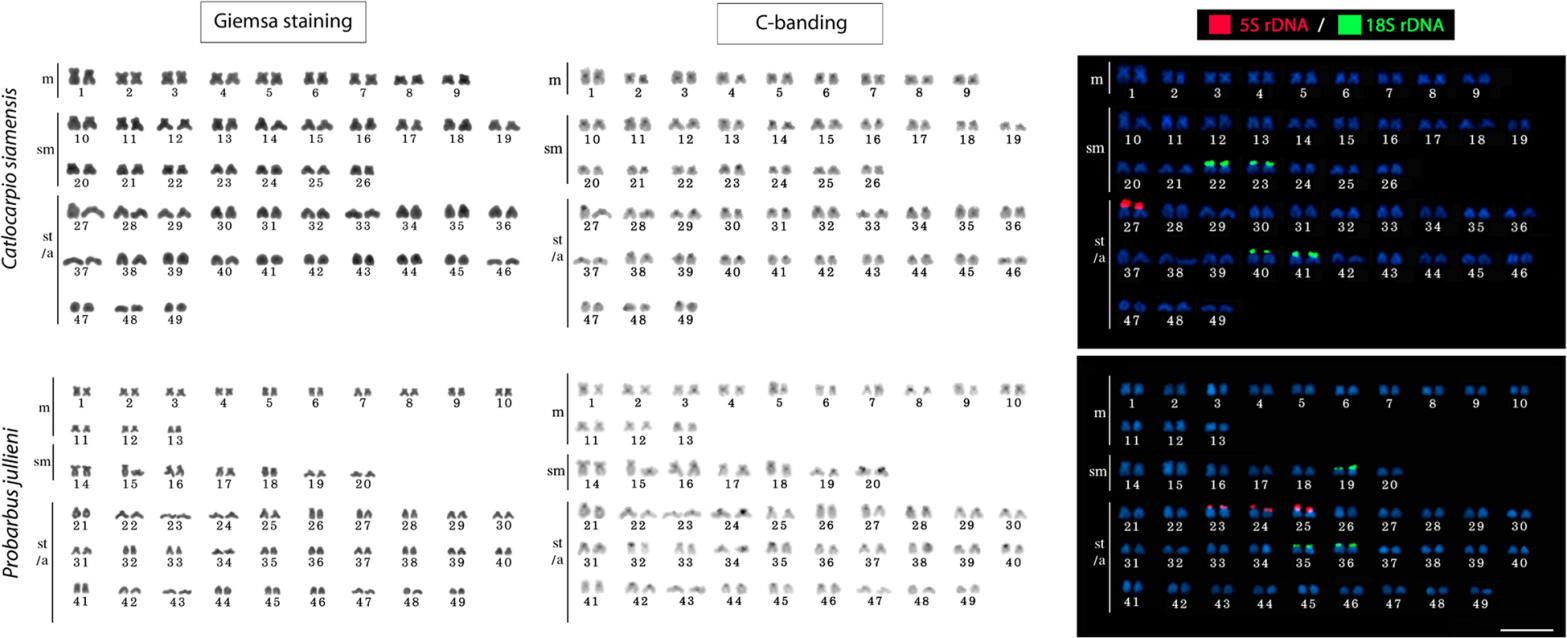
Karyotypes of *Catlocarpio siamensis* and *Probarbus jullieni* arranged from Giemsa-stained, C-banded chromosomes and after dual-colour FISH with 5S (red) and 18S (green) rDNA probes. Scale bar = 5 μm

The mapping of the three short repetitive sequences, (A)_30_, (CA)_15_ and (GA)_15_, showed the same hybridization patterns in both species. While (A)_30_ presented a scattered distribution among all chromosomes, the other two motifs (CA)_15_ and (GA)_15_ are both accumulated in the telomeric regions of several chromosome pairs (Fig. 2).

## Discussion

Despite the sporadic occurrence in higher vertebrates, polyploidy was repeatedly documented across several fish lineages and at different taxonomic levels, implying its independent and recurrent origin [24–27]. Out of the four species placed in the tribe Probarbini, just two – *P. jullieni* and *C. siamensis* – have previously been cytogenetically analyzed. Suzuki and Taki [22] reported for the former species 2n = 98 (18m + 54sm/st + 26a) and the follow-up study from the same authors [23] showed 2n = 98 (18m + 28sm/st + 52a) for the latter species. Our results confirmed 2n for both species but with small differences in the karyotype composition. This incongruence reflects probably the small size of the cyprinid chromosomes, especially those of polyploids. Furthermore, cyprinid chromosomes also exhibit a gradual decrease in size, with the centromere positions ranging step-wisely from median to nearly terminal. Indeed, these features make it difficult to assess the chromosomal categories with accuracy [28–30].

Another important aspect of the comparative cytotaxonomy associated with Probarbini species analyzed herein is their slightly reduced 2n = 98 from the usual 2n = 100 that occurs in the overwhelming majority of the polyploid cyprinids [19]. Representatives of the Schizothoracini and Schizopygopsini tribes, the most ones phylogenetically distant from the Probarbini [9], also display a reduced chromosome number ranging from 90 to 98 [31–38]. In this sense, such 2n reductions seem to be a result of independent evolutionary events. As most of other paleotetraploid cyprinine taxa have 2n = 100, 98 chromosomes found in both species examined here likely represent a derived feature. We suppose that the reduced 2n = 98 might be a shared derived character for species of the whole Probarbini clade. Yet, 2n counts for the two remaining members of this group, i.e. *P. labeamajor* and *P. labeaminor*, remain yet to be determined. An obvious but still unclear question is what evolutionary background stands beyond this 2n reduction? Probarbini are highly potamodromous fishes, while the representatives of the other two lineages are confined to high altitudes in Qinghai–Tibetan plateau, forming small, highly fragmented populations [39]. These lineages also contain hexaploid forms [33] or even triploid derivative of this hexaploid level [40] suggesting complex evolutionary processes.

Rapid changes for content in polyploid genomes represent an integral part of very complex processes leading to both immediate and long-term post-polyploidization alterations, involving restructuring of genome architecture, epigenetic landscape and gene expression patterns. These processes lead to gradual re-diploidization, i.e. re-establishment of genome balance and diploid-like inheritance [41–49]. Repetitive DNA sequences may be either lost during the process of the so-called genome downsizing, or they can be amplified and/or accumulated in gene-poor regions. Such changes may be driven by illegitimate (non-homologous) recombination and deregulated control of (retro) transposition activity [43, 44, 47, 50–52].

Therefore, the mapping of repetitive DNA classes might provide a useful tool for elucidating the dynamics of (especially young) post-polyploid genomes. Alike the comprehensive cytogenetic maps produced for plant neopolyploids e.g. [53–55], similar research in fishes is mainly restricted to mapping of tandemly-repeated clusters of major (nucleolus-forming, 45S) and minor (5S) rDNA classes as they are the most utilized markers in fish cytotaxonomy in general [28, 56–59]. However, studies performing cytogenetic mapping of e.g. several markers such as *Hox* genes or satellite DNAs, in conjunction with recent advances in genome sequencing and bioinformatics, are also starting to be implemented typically in non-teleost acipenserids [60, 61]. Regarding rDNA profiles, they proved useful especially in deciphering the origin of rather recent homoploid or polyploid hybrids, because the intermediate pattern might help to identify the parental species [62–66]. Moreover, if the numbers of rDNA sites remain additive in polyploids of uncertain origin, the mechanism of either auto- or allopolyploidy might be inferred [67–69]. However, both rDNA classes are subjected to dynamic and complementary evolutionary forces leading either to intragenomic sequence homogenization (concerted evolution), or to the emergence of new repeat variants (birth-and-death evolution) [70–76]. In polyploids, these processes are further modified by the nature of polyploidy, with nucleolar dominance [71, 77, 78] and chimeric sequence variants (described also in cyprinids [72–75]) being a typical example found in allopolyploids. Finally, deregulated control of (retro) transposition activity, especially in hybrid genomes, may further greatly contribute to rDNA site number instability/ hypervariability [76], as both rDNA classes are known to be frequent targets for insertion of various mobile elements [79–83]. All these processes are adding complexity to rDNA dynamics and may disable smooth usage of these markers for examination of karyotype differentiation processes. To name a few examples, high polymorphism and/or rapid amplification of rDNA sites were found in homoploid or polyploid cyprinid fishes [29, 62, 84–86] as well as in other paleo- or neopolyploid fish taxa across the teleost phylogeny [58, 87]. However, also some diploid cyprinids might pose a challenge in terms of rDNA site number analysis due to varied degree of intra- or interindividual polymorphism [88, 89] and interspecific variability [90, 91].

In this study, the number and distribution of ribosomal genes were not conserved among both analyzed species. Thus, whereas two and eight 5S and 18S rDNA signals, respectively, were mapped on the chromosomes of *C. siamensis*, a total of six signals for both rDNA classes was observed in the *P. jullieni* karyotype (Fig. 1). Considering only the strictly polyploid taxa, the distribution patterns of the rDNA sites among the cyprinid lineages remain poorly covered [29, 30], except for some representatives of the Torini and Cyprinini tribes. The available data for five Indian species of the genus *Tor* (Torini) [21, 84, 85] demonstrate very similar pattern as found in our present study, i.e., either single or multiple chromosome pairs carrying both ribosomal clusters. However, the genomes of several members of the clade Cyprinini were examined more thoroughly for these markers [19]; also they exhibit variable site numbers for both rDNA classes. As an example, the common carp *Cyprinus carpio*, with a (paleo) tetraploid karyotype 2n = 100, exhibits a re-diploidized pattern for 45S rDNA, with only a single chromosome pair carrying such sites [92, 93]. Also four to eight 5S rDNA loci [94] can be observed there, while in the (paleo-) tetraploid crucian carp *Carassius carassius* the major rDNA sites occupy two different chromosomal pairs, and the minor rDNA encompasses a variable number of 8–18 loci [29, 30]. Besides, a comparable variability of rDNA distribution was also present in several polyploid forms of *C. auratus* complex [63, 67, 95]. While some reports have evidenced co-localized sites of 5S and 45S rDNA clusters in several either diploid or polyploid cyprinids [62, 86, 94, 96], this is not the case for the species studied herein. Finally, the multiple 45S rDNA sites in both Probarbini species display only slight site-number changes, while 5S rDNA shows a re-diploidized condition in *C. siamensis* - accompanied, however, by apparently larger sizes of both homologous loci. Complementary analyses in the remaining Probarbini species, *P. labeamajor* and *P. labeaminor*, will complete the picture of the post-polyploid rDNA dynamics in this clade. However, from the current data, probable mechanisms such as unequal crossing overs, illegitimate recombination and also transpositions might account for the observed patterns, possibly facilitated by the allopolyploid/hybrid origin of the species.

Microsatellites or simple sequence repeats (SSRs) are oligonucleotides of 1–6 base pairs in length, forming excessive tandem repeats of usually four-to-40 units [97–99]. They show abundant distribution throughout eukaryotic genomes, being dispersed or clustered both in euchromatin or heterochromatin. They are highly polymorphic regarding copy number variations [98]. In the fish genomes, microsatellites are usually abundant in the centromeric and telomeric regions, and they can also localize preferentially to sex chromosomes [56, 100, 101]. In our study, both species exhibited the same general hybridization pattern for all applied probes, with the motif (A)_30_ showing moderate abundance and dispersed hybridization pattern throughout the genomes. Otherwise, the dinucleotides (CA)_15_ and (GA)_15_ accumulated exclusively in telomeric and subtelomeric chromosomal regions, corroborating findings from other fish groups studied to date [101–107]. While the bearing of the observed patterns to the dynamics of polyploidy remains unclear, some authors [104, 107] suggested that the preferential targeting of (CA)_15_ and (GA)_15_ and other microsatellite motifs to telomeric and subtelomeric regions might be functionally linked with the structural formation of telomeres. As microsatellites can be found to be embedded within rDNA clusters [108] and all rDNA sites turned out to be terminal in both species under study, we suppose that (CA)_15_ and (GA)_15_ dinucleotides might have contributed to the dynamic behavior of rDNA sites in these Probarbini representatives.

## Methods

### Animals

**Fig. 3:**
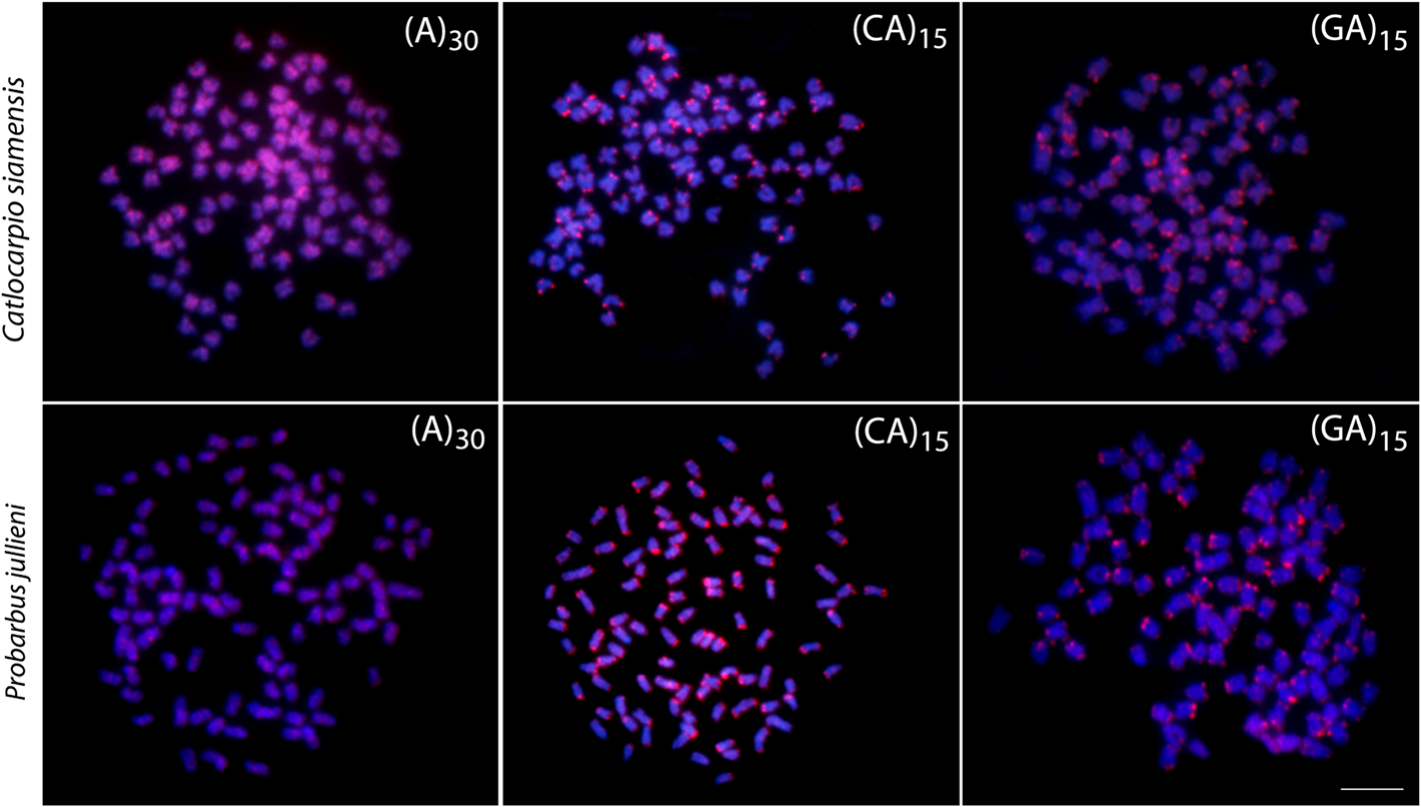
Metaphase plates of *Catlocarpio siamensis* and *Probarbus jullieni* after FISH with different microsatellite motifs. Scale bar = 5 μm

Individuals of *Catlocarpio siamensis* (12♂ and 6♀) and *Probarbus jullieni* (8♂ and 8♀) from different tributaries of the Mekong River basin (Thailand) were analyzed (Fig. 3). The specimens were caught using a hand-net, placed in sealed plastic bags containing oxygen and clean water, and transported to the laboratory. Experiments were performed in accordance with ethical protocols, and anesthesia-using clove oil was administered prior to sacrificing the animals, as approved by the Ethics Committee of Khon Kaen University and by the RGJ committee under no. PHD/K0081/2556 (Thailand). The specimens were deposited in the fish collection of the Cytogenetic Laboratory, Department of Biology Faculty of Science, Khon Kaen University (Thailand).

### Chromosome preparation and C-banding

Mitotic chromosomes were obtained from the anterior kidney, cell suspensions dropped onto microscopic slides and air-dried [109]. Conventional staining was done by 5% Giemsa solution in phosphate buffer (pH 6.8) for 10 min. The distribution of constitutive heterochromatin on chromosomes was demonstrated by C-banding method [110].

### The probe preparation and FISH experiments

Two tandemly arrayed rDNA sequences, namely 5S and 18S, were obtained via PCR from the nuclear DNA of *C. siamensis*. The 5S repeat copy encompassing 120 base pairs (bp) of the 5S rRNA coding region and 200 bp of the non-transcribed spacer (NTS) was produced according to Pendas et al. [111]. The 18S rDNA fragment, with 1,400-bp in length including the 18S rRNA gene coding sequence, was prepared according to Cioffi et al. [112]. Both rDNA fragments were cloned into plasmid vectors and propagated in DH5α *Escherichia coli* competent cells (Invitrogen, San Diego, CA, USA). The final 5S and 18S rDNA probes were directly labeled with SpectrumOrange-dUTP and SpectrumGreen-dUTP, respectively, by Nick translation kit (Roche, Mannheim, Germany) according to the manufacturer’s recommendations. FISH was performed under high stringency conditions following the protocol of Yano et al. [113].

FISH with the set of probes corresponding to three microsatellite motifs (A)_30_, (CA)_15_ and (GA)_15_ was performed as described in [114], with slight modifications. These sequences were directly labeled by Cy3 at the 5′ terminus during synthesis (Sigma, St. Louis, MO, USA).

Chromosomes were counterstained with DAPI (1.2 μg/ml) and mounted in antifading solution (Vector, Burlingame, CA, USA,) in both experiments.

### Image processing

At least 30 metaphase spreads per individual were analyzed to confirm the diploid number, karyotype structure and FISH data. Images were captured using an Olympus BX50 microscope (Olympus Corporation, Ishikawa, Japan) with CoolSNAP and processed using Image Pro Plus 4.1 software (Media Cybernetics, Silver Spring, MD, USA). Chromosomes were classified according to centromere position as metacentric (m), submetacentric (sm) and subtelocentric (st)/acrocentric (a) ones [115], with the st and a chromosome pairs being scored together in one st-a category. For the chromosomal arm number (NF; Nombre Fondamental) to be calculated, m + sm were scored as bi-armed while st + as mono-armed.

## Conclusions

Here we hypothesized that the karyotype characterized by 2n = 98 in both analyzed species might represent a derived character, probably also shared by all members of the Probarbini clade. Besides, we also witnessed considerable changes in the amount and distribution of certain repetitive DNA classes, hence suggesting complex post-polyploidization processes in this small paleotetraploid tribe.

## Abbreviations

2n: Diploid number
a: acrocentric chromosome
DAPI: 4′,6-diamidino-2-phenylindole
dUTP: 2′-Deoxyuridine-5′-Triphosphate
FISH: Fluorescence in situ hybridization
m: metacentric chromosome
NF: Nombre Fondamental
NTS: Non-transcribed spacer
PCR: Polymerase chain reaction
rDNA: ribosomal DNA
rRNA: ribosomal RNA
sm: submetacentric chromosome
st: subtelocentric chromosome
